# Complement Expression and Activation in Osteoarthritis Joint Compartments

**DOI:** 10.3389/fimmu.2020.535010

**Published:** 2020-10-29

**Authors:** Elisa Assirelli, Lia Pulsatelli, Paolo Dolzani, Erminia Mariani, Gina Lisignoli, Olga Addimanda, Riccardo Meliconi

**Affiliations:** ^1^ Laboratory of Immunorheumatology and Tissue Regeneration, Istituto di Ricovero e Cura a Carattere Scientifico (IRCCS) Istituto Ortopedico Rizzoli, Bologna, Italy; ^2^ Department of Medical and Surgical Sciences, Alma Mater Studiorum—University of Bologna, Bologna, Italy; ^3^ Medicine and Rheumatology Unit, IRCCS Istituto Ortopedico Rizzoli, Bologna, Italy; ^4^ Department of Biomedical and Neuromotor Sciences, Alma Mater Studiorum—University of Bologna, Bologna, Italy

**Keywords:** osteoarthritis, complement system, cartilage, synovium, joint tissue

## Abstract

**Objective:**

To investigate complement(C) factors(F) and their activation fragments expression in OA joint tissues.

**Design:**

Immunohistochemistry and quantitative imaging were performed to analyze C3, C4, and CF (factor) B expression on osteochondral biopsies (43 patients) collected during arthroplasty. Isolated chondrocytes and synoviocytes, cartilage and synovial tissues obtained from surgical specimens of OA patients (15 patients) were cultured with or without IL-1β. Real time PCR for CFB, C3, and C4 was performed. Culture supernatants were analyzed for C3a, C5a, CFBa, and terminal complement complex (TCC) production.

**Results:**

In osteochondral biopsies, C factor expression was located in bone marrow, in a few subchondral bone cells and chondrocytes. C3 was the most expressed while factor C4 was the least expressed factor. Gene expression showed that all C factors analyzed were expressed both in chondrocytes and synoviocytes. In chondrocyte cultures and cartilage explants, CFB expression was significantly higher than C3 and C4. Furthermore, CFB, but not C3 and C4 expression was significantly induced by IL-1β. As to C activation factors, C3a was the most produced and CFBa was induced by IL-1β in synovial tissue. TCC production was undetectable in isolated chondrocytes and synoviocytes cell culture supernatants, whereas it was significantly augmented in cartilage explants.

**Conclusion:**

C factors were locally produced and activated in OA joint with the contribution of all tissues (cartilage, bone, and synovium). Our results support the involvement of innate immunity in OA and suggest an association between some C alternative pathway component and joint inflammation.

## Introduction

Local and systemic low-grade inflammation is recognized as one of the major triggering factors in Osteoarthritis (OA) pathogenesis in combination with age, biomechanical stress, and metabolic derangement (1–3). While systemic inflammation is strictly associated with ageing (“inflammaging”) (4) and metabolic alterations, local inflammation is more related to biomechanical stress and traumatic events (5).

Inflammation in OA joints is mainly the result of the activation of the innate immunity response which involves different cells such as macrophages, synoviocytes, chondrocytes, bone cells, and various soluble molecules (6). One of the main molecular pathways in innate immunity is the Complement system, which intervenes in several responses such as leukocyte chemotaxis, microorganism opsonization, phagocytosis, and cell lysis (7).

Complement proteins are synthetized in the liver and by immune cells, but other cell lineages (eg, skin cells, astrocytes, and glia cells, muscles cells) (8) are also involved in physiological and pathological production and activation of complement factors. Complement activation proceeds through three distinct pathways: the classical, the lectin (mannose-activated) and the alternative pathway which converge in the cleavage of C3 component by C3 convertase. This cleavage results in the formation of C5 convertase which in turn activates C5 with the formation of C5b, the first component of C5b-9 complex, known as Terminal Complement Complex (TCC) or Membrane Attack Complex (MAC), responsible for cell lysis (9). However, a large number of non-lytic functions (such as the release of pro-inflammatory cytokines and chemokines or the expression of Platelet-derived or b-Fibroblast growth factors) can result by the generation of C5b-9. During this process, the production of potent inflammatory fragments (C3a and C5a) amplify the inflammatory response by binding to the cognate receptors on several target cells (10).

In addition to its physiological role in infection defense and in removal of tissue damage derived debris, Complement exerts pathological functions in several diseases characterized by chronic inflammation. It is well known its role in inflammatory arthritides and connective tissue disorders, while more recently complement activation has been involved in OA pathomechanisms (5, 10–13).

In this study we aimed at elucidating the different contributions of OA patient joint tissues in the production of alternative and classical Complement pathway factors and at evaluating any local activation of Complement. Thereof, the central molecule C3, the classical pathway molecule C4 and the alternative pathway molecule Factor B (CFB) were assessed in different tissue compartments of OA patient joints. Then the activation fragments generated in the same tissues as well as C5b-C9 complex and the role of IL-1β in these processes were also evaluated.

We found that CFB was the most expressed component followed by C3, that the activation fragments C3a, C5a, and CFBa were produced *in vitro* mainly by isolated chondrocytes and that TCC was mainly produced by cartilage tissue and to a lesser extent by synovial tissue.

## Materials and Methods

This study was approved by the ethical committee of the Rizzoli Orthopaedic Institute and written informed consent was obtained from the patients. The procedures were in accordance with the ethical standards of the responsible committee on human experimentation (institutional and national) and with the Helsinki Declaration of 1975, as revised in 2013.

### Osteochondral Biopsies: Histologic Score and Immunohistochemistry Analysis of C3, C4, and CFB

Full-thickness osteochondral biopsies were collected (14) from 43 patients, 16 with OA (9 males, 7 females; mean age, 67 years; range, 59–75 years), 12 with Rheumatoid Arthritis (RA) (12 females; mean age, 65 years; range, 22–85 years) and 15 Post-traumatic patients (PT) (7 males, 8 females; mean age, 64 years; range, 24–82 years), undergoing knee, hip or shoulder arthroplasty.

Briefly, osteochondral biopsies were fixed, embedded in paraffin, sliced in serial sections (5 µm thick) by a microtome and stained with hematoxylin-eosin and Safranin O fast green, as previously described (15).

The severity of cartilage damage was assessed using Mankin score evaluation (16) and immunohistochemistry analysis was used to evaluate C3, C4 and CFB positive cells across cartilage and bone. Sections were heated overnight at 56°C, rehydrated, and incubated with primary antibodies (Santa Cruz biotech, Dallas, USA) against C3 (at a concentration of 2.5 µg/ml), C4 (at a concentration of 0.5 µg/ml) and CFB (at a concentration of 12.5 µg/ml). Signals were developed with a biotin/streptavidin amplified, alkaline phosphatase-based detection system (Biocare Medical, Pacheco, CA, USA) with fuchsin as a substrate. After nuclear counterstaining with hematoxylin, sections were mounted in glycerol gel and stored for subsequent analysis. Sections of each biopsy were processed as negative controls, according to the above-described procedure, omitting the primary antibody. Specificity was assessed by appropriate isotypic control at the concentration of the corresponding primary antibody.

Semi-quantitative image analysis of immunohistochemistry stained slides was performed on optical microscope fields (20× objective lens) for each section. The analysis was performed using Red/Green/Blue (RGB) with Software NIS-Elements and Eclipse 90i microscope (Nikon Instruments Europe BV) equipped with a CCD camera (dimension of the sensor 2/3 inches) 104 mounted on 0.7× C-mount. Imaging analysis results were expressed as percentages of positive area in the section analyzed.

### Tissue Explants

Ex vivo cartilage and synovium tissue explants were obtained from 15 patients with knee OA (6 males, 9 females; mean age, 72 years; range, 60–83 years) undergoing joint replacement surgery. Cartilage and synovium tissues were weighted and seeded in 24-well plates, one specimen per well.

#### Chondrocyte Isolation

Chondrocytes were isolated by sequential enzyme digestion after cartilage fragmentation as previously detailed (17) and incubated in DMEM medium (SIGMA, Sigma Aldrich, St. Louis, USA) supplemented with 100 U/ml penicillin, 100 µg/ml streptomycin (Invitrogen, Carlsbad, CA, USA) 10% FBS (GIBCO, Thermo Fisher Scientific, NY, USA) for 24 h at 37°C with 5% CO2 in a humidified atmosphere. High-density chondrocyte cultures were seeded at 250,000 cells per well in 24-well plates.

#### Synoviocyte Isolation

Synovial membrane specimens were finely minced. Tissue fragments were seeded in petri dishes and maintained with OPTIMEM (GIBCO, Thermo Fisher Scientific, NY, USA) culture medium supplemented with 100 U/ml penicillin, 100 µg/ml streptomycin (Invitrogen, Carlsbad, CA, USA), 10% FBS (GIBCO, Thermo Fisher Scientific, NY, USA) for about 10 days at 37°C with 5% CO2 in a humidified atmosphere (18). The cells were then maintained into culture flasks and all experiments were performed on cells obtained between the third and fifth passage. Synoviocytes were seeded at 200,000 cells per well in 24-well plates.

#### In Vitro Stimulation

Tissue explants and isolated cells were all maintained with appropriate culture medium prepared as described above, but without serum (starvation conditions) for 24 h and then they were stimulated with 2 ng/ml of rhIL-1β (R&D Systems, Minneapolis, USA). Optimal IL-1β concentration and incubation time for detecting complement factor production were previously determined by dose-dependent and kinetic experiments (not shown). After 24 h of incubation, culture supernatants (both unstimulated and stimulated) were collected and maintained at −80°C until analysis. Cartilage tissue explants and isolated chondrocytes were analyzed to elucidate the role of cartilage matrix components and the ability of isolated chondrocytes to produce complement factors. Synovial tissue (comprising both synovial macrophages and fibroblast like synoviocytes-FLS) and isolated FLS alone were analyzed to discriminate the contribution of both cell type to complement production. RNA was extracted from isolated cells by a direct lysis in the culture plates. Tissue explants were frozen and maintained in liquid nitrogen until RNA extraction. For this purpose, frozen samples were pulverized with the grinding mill Mikro-Dismembrator S (Sartorius Stedim Italy SpA, Italy) in 5 ml PFTE shaking flasks with a stainless-steel grinding ball.

#### Real-Time, Quantitative Reverse Transcriptase Polymerase Chain Reaction (RT-PCR)

Total cellular RNA was extracted using TRIZOL reagent (INVITROGEN, Life Technologies, NY, USA) following the protocol recommended by the manufacturer. RNA was reverse transcribed using SuperScript VILO cDNA Synthesis kit (INVITROGEN, Life Technologies, NY, USA), following manufacturer’s instructions. RNA specific primers for PCR amplification (**Table 1**) were generated from GeneBank sequences using the NCBI primer-Blast tool. Real-time PCR was run on the LightCycler Instrument (Roche S.p.A, Monza, Italy) using the SYBR Premix Ex Taq (TAKARA Biomedicals; Tokyo, Japan) with the following protocol: 95°C for 10 s, followed by 45 cycles at 95°C for 5 s and 60°C for 20 s and the increase in PCR product was monitored for each amplification cycle by measuring the increase in fluorescence due to the binding of SYBR Green I Dye to dsDNA. The crossing point values were determined for each sample and specificity of the amplicons was confirmed by melting curve analysis. Amplification efficiency of each amplicon was evaluated using 10-fold serial dilutions of positive control cDNAs and calculated from the slopes of log input amounts plotted versus crossing point values. They were all confirmed to be high (>92%) and comparable; mRNA levels for each target gene were calculated normalized (ratio) to glyceraldehyde-3 phosphate dehydrogenase (GAPDH, reference gene), according to the ΔΔCt method and expressed as “Number of molecules per 100,000 GAPDH”.

**Table 1:** List of primers used in Real-Time PCR.

Gene		Primer Sequence	Ta (°C)
*Gapdh*	Forward	CCTGGCCAAGGTCATCCATG	60
	Reverse	CGGCCATCACGCCACAGTT	
*C3*	Forward	TCAACCACAAGCTGCTACCC	60
	Reverse	CTGGCCCATGTTGACGAGTT	
*CFB*	Forward	GGGGTAGAGATCAAAGGCGG	60
	Reverse	TGGATTGCTCTGCACTCTGC	
*C4*	Forward	GGGTTTGGTGGGCAATGATG	60
	Reverse	GAGGCTTCCACTCTCTGCTT	

#### Complement Activation Fragment Concentrations

Complement fragment C3a, C5a, CFBa, and complex C5b-9 concentrations were evaluated in culture supernatants obtained both from tissues and isolated chondrocytes and synoviocytes by using commercial kits (C3a and C5b-9 Elabscience E.L.I.S.A. kit, Houston, TX, USA; C5a Origene E.L.I.S.A. kit, Rockville, MD, USA; CFBa Quidel E.L.I.S.A. kit, San Diego, CA, USA) following the manufacturer’s instructions. Factor concentration was normalized for milligram (mg) of tissue or for 100,000 cell number as appropriate.

### Statistical Analysis

Data were expressed as medians, interquartile ranges and minimum to maximum values. The Kolmogorov Smirnov test was performed to test normality of continuous variables. The Friedman ANOVA test with Dunn’s correction was used to compare Complement factor staining positivity within each disease and Kruskal-Wallis test with Dunns correction was performed to compare a single Complement factor among diseases. The Spearman Correlation test was used to assess the correlation between the staining positivity of each complement factor and the Mankin score. The General Linear Mixed Models analysis with Complement factors as dependent variables, articular tissue types and IL-1β stimulus as fixed effects and age as covariate were used to assess the influence of articular tissue type and IL-1β stimulus alone or combined on modifications of the analyzed factors. Wilcoxon matched paired test with Bonferroni correction for multiple comparisons was used to compare the production of complement factor activation fragments. A p value <0.05 was considered significant. Statistical analysis was performed using SPSSv.19.0 (IBM Corp., Armonk, NY, USA) and GraphPad prism for Windows, version 5.01 (Nashville, TN, USA).

## Results

### C3, C4, and CFB Staining on Osteochondral Biopsies

**Figure 1:**
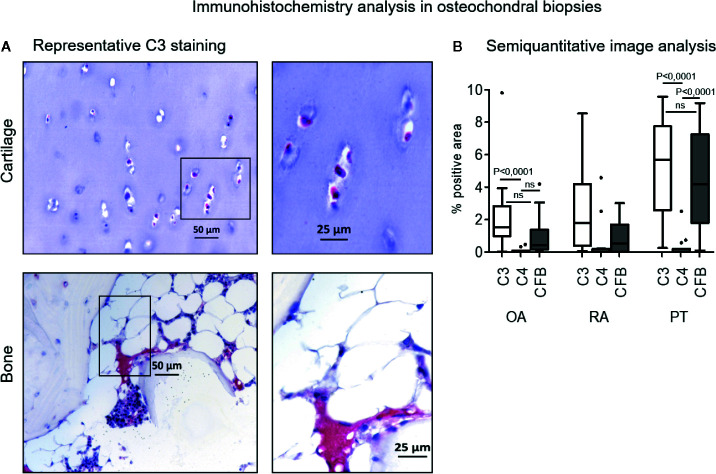
Immunohistochemistry analysis of C3, C4, CFB in osteochondral biopsies. **(A)** Bone and cartilage C3 representative staining positivity (original magnification, 10× scale bar, 50 µm and 20×; scale bar, 25 µm). Red staining for positive cells, counterstaining with hematoxylin in purple. **(B)** Image analysis of C3, C4 and CFB in osteochondral biopsies: Osteoarthritis (OA), Rheumatoid Arthritis (RA), Post Traumatic cases (PT). Results are expressed as median (bars), interquartile ranges (boxes), minimum to maximum values (whiskers) and outliers (solid circles). Comparison among C factors within patient groups showed statistical significance in OA patients (C3 vs C4, p<0.0001) and PT patients (C3 vs C4 and C4 vs CFB, p<0.0001). Comparison among patient groups within C factors. C3 factor: OA vs RA and RA vs PT, not significant; OA vs PT, p<0.05. C4 factor: OA vs RA, RA vs PT and OA vs PT, not significant. CFB factor: OA vs RA, not significant; RA vs PT and OA vs PT, p<0.05.

Complement factors staining was mainly located in the bone marrow, with positive areas widespread among bone trabeculae and adipose tissue, whereas cartilage positivity was found only in few samples (**Figure 1**). Semi-quantitative image analysis of immunohistochemical staining showed that C3 was more expressed than C4 in OA and PT patients (C3 vs C4 p<0.0001 either in OA and PT). In PT patients also CFB was more expressed than C4 (p<0.0001). No difference was observed between C3 and CFB staining in OA and PT. In RA patients, even if the expression trend of C3, C4 and CFB appeared similar to that observed in OA and PT groups, no difference among these factors was observed.

Both C3 and CFB expressions were significantly augmented in PT compared to OA patients (p< 0.05), furthermore CFB was also more expressed in PT than in RA patients (p<0.05) (**Figure 1**). C3 and CFB were similar in both OA and RA, whereas C3 was similar in RA and PT.

C4 expression appeared almost negligible, except some sporadic cases, and similarly expressed in all patient groups. Complement factors expression did not correlate with Mankin score, as determined by Spearman Correlation test and no differences were found among different joints (data not shown).

### Complement Factor Gene Expression in Cartilage Tissue, Chondrocytes, and Synoviocytes

**Figure 2:**
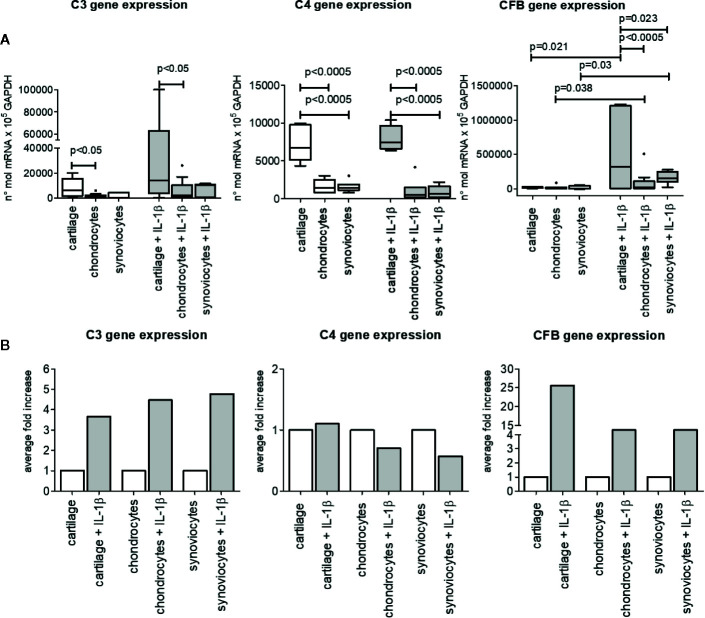
C3, C4, and CFB gene expression in OA cartilage tissue, isolated chondrocytes and isolated synoviocytes. **(A)** Relative amount of gene expression. Results are expressed as median (bars), interquartile ranges (boxes), minimum to maximum values 542 (whiskers) and outliers (solid circles). Only statistically different comparisons where indicated. **(B)** Average fold increase in respect to unstimulated samples. Bars indicates average mean.

Cartilage tissue as well as isolated chondrocytes and synoviocytes spontaneously expressed complement factor genes of both the classical and alternative ways, even if in different amounts (**Figure 2**).

IL-1β pro-inflammatory stimulus enhanced only CFB gene expression, either in cartilage tissue (p=0.021) and in isolated chondrocytes and synoviocytes (p=0.038 and p=0.030 respectively). On the contrary, C3 and C4 basal gene expression was not up-modulated by IL-1β stimulation (**Figure 2**).

Furthermore, the influence of tissue type on gene expression of the three analyzed factors in both unstimulated and IL-1β–stimulated conditions was observed.

Indeed, C3 gene basal expression as well IL-1β–stimulated one was higher in cartilage tissue than in isolated chondrocytes (p<0.05), whereas no difference was found between cartilage tissue and isolated synoviocytes both in basal and stimulated conditions (**Figure 2**).

As to C4 gene, besides its different expression between cartilage and isolated chondrocytes (p<0.0005, both in unstimulated and stimulated conditions), it also showed a greater expression in cartilage than in isolated synoviocytes (p<0.0005, both in unstimulated and stimulated conditions).

No difference in C3 and C4 gene expression was observed between isolated chondrocytes and synoviocytes, whatever unstimulated or stimulated.

CFB gene expression was similar in all types of unstimulated samples analyzed (**Figure 2**), on the contrary under the IL-1β–stimulated conditions, CFB gene was more expressed in cartilage tissue than in isolated chondrocytes (p<0.0005) and synoviocytes (p=0.023). No CFB gene differences were observed between isolated chondrocytes and synoviocytes stimulated with IL-1β (**Figure 2**).

The comparison among C3, C4 CFB gene expressions to evaluate the most expressed factor independently of articular compartment, as determined by General Linear Model analysis, evidenced that CFB was the most expressed factor, followed by C3 and by C4 (CFB vs C3 and vs C4, p<0.0005; C3 vs C4, p<0.02), (data not shown).

### Release of Activation Fragments in Culture Supernatants of Cartilage and Synovium Tissues and of Isolated Chondrocytes and Synoviocytes

**Figure 3:**
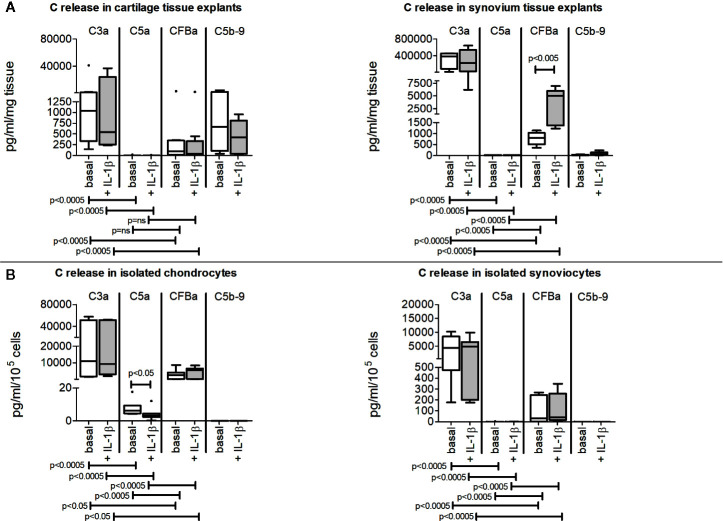
Release of C3a, C5a, CFBa activation fragments and C5b-9 complex from OA patients. Results are expressed as median (bars), interquartile ranges (boxes), minimum to maximum values (whiskers) and outliers (solid circles). Where not indicated, the comparisons are not significant. **(A)** Cartilage and synovium tissue culture supernatants. **(B)** Isolated chondrocyte and synoviocyte culture supernatants.

Cartilage and synovium tissues (**Figure 3A**), isolated chondrocytes and synoviocytes (**Figure 3B**) were all able to spontaneously release complement activation fragments (C3a, C5a, CFBa) in culture supernatants, even if at very variable concentrations.

C3a was the most produced among activation factors independently of the sample type (**Figures 3A, B**) followed by CFBa, whereas C5a release was the lowest.

On the contrary, C5b-9 complex release was only detectable in culture supernatants of cartilage and synovium tissues (**Figure 3A**) but was below the detection limit when evaluated in culture supernatants obtained from isolated chondrocytes and synoviocytes (**Figure 3B**).

IL-1β did not influence activation fragment release but the CFBa up-modulation in synovium tissue (p=0.002) (**Figure 3A**) and for C5a down-modulation in isolated chondrocytes (p=0.038) (**Figure 3B**).

**Figure 4:**
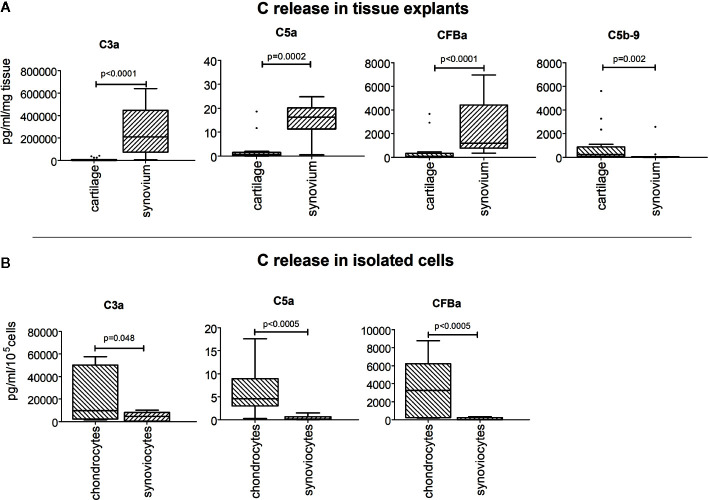
Comparison of C3a, C5a, CFBa fragment release between cartilage and synovium tissues **(A)**, and between isolated chondrocytes and synoviocytes **(B)**. Results are expressed as median (bars), interquartile ranges (boxes), minimum to maximum values (whiskers) and outliers (solid circles).

The comparison of the release of C3a, C5a, CFBa fragments between cartilage and synovium tissues, as determined by General Linear Model analysis, showed that all these factors were more concentrated in synovium tissue supernatants (p<0.0002 at least) (**Figure 4A**). Conversely, the results obtained by comparing isolated cells showed higher release of activation fragments by isolated chondrocytes (p<0.05) (**Figure 4B**).

## Discussion

In this study, the contribution of the different joint tissues (cartilage, synovium and bone) in producing components of the Complement system was evaluated. We found that C3, C4, and CFB complement factors were expressed by all joint tissues.

Immunohistochemical staining in osteochondral biopsies from OA, RA and PT patients showed a similar pattern, with C3 and CFB more expressed than C4. This expression was mainly located in the bone marrow portion of the osteochondral biopsies, where it could contribute to sustain bone inflammation and bone marrow edema, that is one of the pathological hallmark of OA (19).

A newly found potential trigger of innate immunity in OA is Gut Microbioma (GM). In a very recent study, Dunn and colleagues provided the first evidence of microbial nucleic acid signatures in human and mouse cartilage tissue. Interestingly they found an increase in gram-negative constituents in OA cartilage compared to control cartilage (20) GM was firstly detected in synovial fluid of OA patients, but it is hypothesized that it could reach subchondral cartilage through blood vessels located in subchondral bone tide mark, at the interface between bone and cartilage. In fact, osteochondral plate angiogenesis occurring early in OA could facilitate transient migration of still living bacteria or some of their products up to the deeper layers of cartilage (21). Bacterial DNA is strongly immunogenic and together with lipopolysaccharide produced by gram-negative constituents can prime the proinflammatory innate immune response in joints, activating innate immunity both through Toll Like Receptors (which are up-regulated in OA) and complement system activation. Complement factors were more expressed in osteochondral biopsies from PT patients, in agreement with described trauma-related activation of complement cascade due to the release of damage-associated molecular patterns (DAMPs) by injured tissues (22). Since trauma is an event potentially leading to OA development, it would be interesting, in light of these results, to analyze post-traumatic patients that have actually developed OA, also in relation to the expression level of complement factors.

Gene expression in cartilage tissue and isolated chondrocytes and synoviocytes from OA knees, confirmed the immunohistochemical results: C3 and in particular CFB were more expressed than C4, thus demonstrating a major *in situ* generation of the main molecules involved in alternative complement activation pathway.

In addition, the prevalent expression of C3 and CFB genes in cartilage tissue compared to isolated chondrocytes and synoviocytes, supports the hypothesis that endogenous extracellular matrix (ECM) components are able to stimulate Complement factor expression.

Cartilage oligomeric matrix protein (COMP), a family of extracellular matrix proteins, also known as thrombospondin-5 (TSP-5), is recognized as particularly relevant in OA pathogenesis. Indeed, COMP serum concentrations were found increased in patients with early signs of cartilage damage (23) and correlated with synovial fluid concentrations (24). In addition, COMP serum levels were associated with the development of pain and radiographic knee signs of OA (25). Interestingly, COMP exerts a dual effect on the complement cascade as it can inhibit both the classical and lectin pathways by binding C1q and, at the same time, it can activate the alternative pathway by binding C3 and properdin. Finally, elevated levels of COMP-C3b complex has been found in OA synovial fluid (26, 27).

Besides COMP, other cartilage ECM components can interact with complement factors or activate complement cascade such as: Collagen II-containing immune-complexes (28), Hyaluronan intra-articular therapeutic injections (29) and DAMPs derived from OA tissue debris (30).

The different amounts of C3a, C5a, CFBa activation fragments detected in cartilage and synovium tissues as well as in isolated chondrocyte and synoviocyte cultures evidenced that Complement activation may take place without the need for parent molecules coming from blood stream.

We found higher production of activation fragments in culture supernatants of synovium tissue compared to cartilage one, whereas the opposite was observed in culture supernatants of isolated chondrocytes and synoviocytes. In these conditions higher concentration of complement activation fragments was produced by isolated chondrocytes, in agreement with previous observations demonstrating that complement proteins were synthesized locally by chondrocytes and upregulated in OA joint (12).As to the conflicting results on synovium when analyzed as a whole tissue or as isolated synoviocytes, it must be considered that synovium tissue comprises specialized resident Fibroblast-Like Synoviocytes (FLSs) that are interspersed with macrophages (31).

Accordingly, we can speculate that the difference in complement activation fragment production may due to the progressive loss of the macrophage component in cultures of isolated synoviocytes and the resulting FLS enrichment, due to *in vitro* passages (32). If, on the one hand, macrophages, as part of the innate immune system arm, were already known to produce complement factors (33) on the other, to the best of our knowledge, this study first showed that human primary fibroblast-like synoviocytes from OA patients also contributed to C activation factors production, in line with their known role in synovitis (34). However, it cannot be excluded that the normalization of the results by weight of tissue, not taking in account the cellularity characteristics between cartilage and synovium, may have caused an overestimation in the whole synovium.

In addition, C3a and C5a are also involved in the onset of pain (10) and in our model a greater release of C3a than C5a was observed, suggesting a major C3a responsibility in the development of pain. If antagonists for the respective receptors are possible candidates for the treatment of this symptom, the blockade of the C3a fragment receptor could probably be more promising, considering also C3 key position at the crossing between classical and alternative Complement pathways (35, 36). However, since C3a and C5a are also chemotactic for bone marrow–derived hematopoietic stem cells and mesenchymal stem cells (MSC) (37–39), the inhibition of specific receptors could prevent MSC trafficking, thus negatively influencing OA cartilage repair and bone remodeling (40, 41).

Similarly to single activation factors, also C5b-9 complex displayed paradoxical results being present only in tissue cultures (both cartilage and synovium), suggesting that extracellular matrix components are also able to influence the Terminal-Complement Complex formation as evidenced for the initial steps of the Complement cascade.

TCC is important in mediating chondrocyte death in OA or (in sub-lytic amount) in activating signaling pathways that drive the expression of catabolic and pro-inflammatory molecules (10). In particular, it increases the chondrocytes’ expression of multiple genes encoding cartilage-degrading enzymes (MMPs- and ADAMTSs), inflammatory cytokines and chemokines as well as the expression of other complement effectors. Therefore, both cartilage and synovium tissues can synergistically produce Complement activation factors, thus amplifying pathogenic complement signaling in osteoarthritis (42).

As concerning the effect of IL-1β stimulation, we noted that its action favored the alternative way of Complement by up-modulating both CFB gene expression in all experimental settings and CFBa fragment release in synovium tissue.

Concurrently, IL-1β decreased C5a fragment production in isolated chondrocytes cultures but was totally irrelevant for the other Complement activation fragments analyzed. IL-1β is a pleiotropic cytokine involved in many inflammatory pathways and it is considered an important player in cartilage degradation although the exact contribution of IL-1β to joint destruction *in vivo* remains controversial. Indeed, a recent study showed the non-involvement of IL-1β in synovial inflammation and cartilage destruction during collagenase-induced OA and it was suggested that other inflammatory mediators (possibly the alarmins, IL-6 and IL-17) could be responsible for the joint damage (43, 44). In agreement, in a recent study performed in a translational model, complement factor B was upregulated in IL-17A–treated cartilage explant (45).

Finally, Osteoarthritis treatment with IL-1β inhibitors was not fully satisfactory, thus resizing the role of this interleukin in OA pathogenesis (46–48). Lastly, we cannot exclude that the different response to stimulation with IL-1β in the analyzed tissues may also in part depend on the various distribution of IL-1 receptors in cartilage, synovium and isolated chondrocytes and synoviocytes (49).

Overall the results of this study indicate a higher expression of factors belonging to alternative Complement Pathway. A pivotal role of the alternative way was suggested in chondrocyte transformation and terminal differentiation during endochondral ossification (a fundamental step in OA progression, leading to osteophyte formation), based on the localization of C3, CFB and properdin (a positive regulator of complement alternative pathway) in resting and in hypertrophic zone of cartilage (50).

Furthermore C3f, a fragment released by catabolic degradation of C3b by Factor H, a regulator of alternative pathway of complement (51) has been identified as specific biomarker for OA, pointing out the prevailing involvement of the Complement alternative way in Osteoarthritis.

Recently, the role of the alternative pathway was validated by the demonstration of a local expression of adipsin (a component of the alternative complement way) in OA joint tissues and by the association between adipsin and synovial membrane inflammation and cartilage damage through the activation of complement (52). In conclusion, this study concurs to the accumulating evidences concerning the involvement of the innate immune response in the pathogenesis and progression of OA and suggests an association between some C alternative pathway component and joint inflammation, possibly suggesting complement as a future therapeutic target for patients with Osteoarthritis.

## Data Availability Statement

The datasets generated for this study are available on request to the corresponding author.

## Ethics Statement

The studies involving human participants were reviewed and approved by Rizzoli Orthopedic Institute ethic committee, IRCCS Istituto Ortopedico Rizzoli, Bologna, Italy. The patients/participants provided their written informed consent to participate in this study.

## Author Contributions

All authors contributed to the article and approved the submitted version. EA, LP, and RM conceived and designed the study. EA, PD, and OA performed the experiments. LP, EA, EM, and RM drafting of the article. GL and EM critically revised the article for important intellectual content.

## Funding

This research was funded by Italian Health Ministry “5 per mille funds” and by Bologna University RFO funds.

## Conflict of Interest

The authors declare that the research was conducted in the absence of any commercial or financial relationships that could be construed as a potential conflict of interest.
